# Conjugative Selectivity of Plasmids Is Affected by Coexisting Recipient Candidates

**DOI:** 10.1128/mSphere.00490-18

**Published:** 2018-12-19

**Authors:** Ayako Sakuda, Chiho Suzuki-Minakuchi, Kazunori Okada, Hideaki Nojiri

**Affiliations:** a Biotechnology Research Center, The University of Tokyo, Tokyo, Japan; b Collaborative Research Institute for Innovative Microbiology, The University of Tokyo, Tokyo, Japan; University of Iowa

**Keywords:** Gram-negative bacteria, *Pseudomonas*, conjugation, plasmids

## Abstract

Understanding the mechanisms underlying plasmid behavior under conditions of various environments is important to predict the fate of plasmids in nature. Most previous studies on plasmid transfer employed two strains: one as a donor and the other as a recipient. However, in natural environments, there are usually different recipient cells available to which plasmid can be transferred. In this study, to reveal the underlying mechanisms, we assessed the transferability of plasmids from one donor strain to either of two recipient candidates as the most simplified model. We used Pseudomonas putida KT2440 and Pseudomonas resinovorans CA10dm4 as model hosts and pCAR1 (IncP-7), NAH7 (IncP-9), pB10 (IncP-1β), and R388 (IncW) as model plasmids. As expected, in most cases these plasmids were generally transferred more frequently to a recipient of the same species than to a recipient of a different one under conditions of liquid and filter mating, although NAH7 was transferred from P. resinovorans more frequently to P. putida than to P. resinovorans during filter mating. With the exception of pCAR1, which was less affected, the coexistence of other recipients enhanced the preferences of conjugative transfer to the same species. In particular, preferences corresponding to transfer from P. putida to a different recipient (P. resinovorans) were reduced by the presence of a coexisting same recipient (P. putida) during transfer of NAH7 in liquid and transfer of R388 in filter mating. We determined that large cell aggregates and substances secreted into culture supernatant were not responsible for this phenomenon. Overall, the results of this study suggest the existence of unknown factors determining optimal plasmid transfer to native recipients.

**IMPORTANCE** Most previous studies on plasmid conjugal transfer employed experimental setups with two strains: one as a donor and the other as a recipient. However, the results obtained sometimes failed to agree with observations obtained under natural environmental conditions or in a model microcosm using natural soil and water samples. Therefore, we consider that there is a “gap” in our understanding of plasmid behavior in the context of bacterial consortia that exist under the actual environmental conditions. In this study, we clearly showed that the conjugation selectivity of a plasmid can be affected by the recipient candidates existing around the donor strain by the use of a simplified experimental setup with one strain as the donor and two strains as recipients. These phenomena could not be explained by factors known to affect plasmid transfer as suggested by previous studies. Therefore, we suggest the presence of novel elements regulating plasmid transfer within consortia.

## Abstract (precis)

Most previous studies on plasmid conjugal transfer employed experimental setups with two strains: one as a donor and the other as a recipient. However, the results obtained sometimes failed to agree with observations obtained under natural environmental conditions or in a model microcosm using natural soil and water samples. Therefore, we consider that there is a “gap” in our understanding of plasmid behavior in the context of bacterial consortia that exist under the actual environmental conditions. In this study, we clearly showed that the conjugation selectivity of a plasmid can be affected by the recipient candidates existing around the donor strain by the use of a simplified experimental setup with one strain as the donor and two strains as recipients. These phenomena could not be explained by factors known to affect plasmid transfer as suggested by previous studies. Therefore, we suggest the presence of novel elements regulating plasmid transfer within consortia.

## INTRODUCTION

Plasmids are mobile genetic elements that facilitate rapid adaptation to environmental changes and subsequent evolution of bacteria by conjugal transfer in natural environments (1). Many factors affect plasmid transfer. For instance, the transcription of transfer genes on plasmids is regulated by a host factor(s). There is a cross talk between the plasmid and the host chromosome during transcriptional regulation of transfer genes carried on the F plasmid (2, 3). Some recipient factors, such as the clustered regularly interspaced short palindromic repeat (CRISPR)-Cas system, are known to degrade foreign DNA. Richter et al. suggested that acquisition of a new spacer by the type I-F CRISPR-Cas system resulted in curing of the plasmid and that increasing the number of spacers reduced the conjugation efficiency (4). Surface exclusion and entry exclusion also inhibit the invasion of foreign DNA into recipient cells (5). Moreover, the outer membrane protein OmpA and lipopolysaccharides (LPSs) of the recipient cell are required for efficient conjugation of the F plasmid (6, 7). It has been suggested that PilV adhesin, which is thought to be located at the tip of thin pili, determines recipient specificity during liquid mating of the antibiotic-resistant IncI1 plasmid R64 through recognition of LPSs on the surface of recipient cells (8). It is also known that the nitrogen-related phosphotransferase system (PTS^Ntr^) in Pseudomonas putida is involved in inhibiting the conjugation efficiency of the naphthalene degradative IncP-9 plasmid NAH7 (9, 10) from Escherichia coli (11). The combination of donor and recipient has been found to be responsible for the plasmid conjugation efficiency, with an enhanced tendency of more-frequent plasmid transfer to the same species (12). On the other hand, genome-wide screening in E. coli failed to identify the essential factor necessary for conjugation of the antibiotic resistance IncW plasmid R388 (13, 14) on the recipient chromosome (15). Still, the mechanism for recognition of the recipient cell and the factors that determine conjugation host range remain to be clarified.

Almost all of these factors were determined in studies performed under laboratory conditions. However, considering the differences between laboratory and natural environmental conditions, it is important to clarify the behaviors of plasmids and their hosts under natural conditions. Comparing plasmid behaviors among different hosts or under different conditions enables us to predict the fate of plasmids in natural environments. Many environmental factors such as temperature, nutrient availability, and high-salt stress can affect plasmid behavior (16, 17). It is also known that the peptide pheromone cCF10 facilitates cell aggregation and enhances the transfer frequency (TF) of the antibiotic resistance plasmid pCF10 in Enterococcus faecalis, which is a Gram-positive bacterium (18, 19). In the case of Gram-negative bacteria, it has been known that some compounds in the cell culture such as fatty acids can affect the transfer ability of IncF, IncW, and IncH plasmids (20). Similarly, quorum-sensing systems regulate the transfer of Ti plasmid in Agrobacterium tumefaciens (21). It has also been demonstrated that coresidential plasmids in the same host cell can affect each other’s conjugation efficiencies (22–24). Moreover, our studies using carbazole-degradative IncP-7 plasmid pCAR1 (25–27) as a model also suggested that some environmental factors can affect plasmid conjugation. We showed that the conjugation efficiency of pCAR1 is promoted by the divalent cations Ca^2+^ and Mg^2+^ (28, 29). Furthermore, differences in cell density and mating conditions (liquid mating or filter mating) affected the plasmid conjugation efficiency of pCAR1 (30). Furthermore, in an artificial microcosm study using 15 different bacterial strains, including seven *Pseudomonas* strains, conjugative transfer of pCAR1 was detected only to Pseudomonas resinovorans. In contrast, pCAR1 conjugation to other *Pseudomonas* strains could be detected in filter mating experiments using one donor and one recipient strain (31, 32). These results indicated that the conjugation host range of the plasmid can be affected by the surrounding environment. To clarify the factor(s) responsible for these phenomena, we had to employ a simplified experimental setting.

Conjugation occurs among bacterial consortia under natural conditions. There are several types of candidate recipient cells around the donor cell when conjugation occurs. In most of the studies described above, the mating experiments were performed by combining one donor strain and one recipient strain (1:1 mating), which does not reflect the actual natural environmental conditions, in which there are several types of strains present around the donor strain at the same time. Therefore, in this study, we used two different species as possible candidate recipients and one donor strain (1:2 mating) as a most extensively simplified conjugation design under natural conditions. We employed P. putida KT2440 (33) and P. resinovorans CA10dm4 (pCAR1-cured derivative strain of CA10) (34) as model hosts and used pCAR1, NAH7, antibiotic resistance IncP-1β plasmid pB10 (35), and antibiotic resistance IncW plasmid R388 as model plasmids to perform liquid and filter mating assays. Using this 1:2 mating system, we evaluated the effect of a coexisting candidate recipient on transconjugant formation by conjugation. We evaluated the transconjugant formation efficiency of each plasmid by TF (calculated by dividing the CFU per milliliter of transconjugant cells by the CFU per milliliter of donor cells).

## RESULTS

### Liquid mating experiments.

In order to construct the experimental setup of mating assay, we first determined the optimal time for detection of the effect of a coexisting candidate recipient on transconjugant formation. Mating was performed for 1 h, 3 h, and 16 h. As shown in Fig. S1 in the supplemental material, the tendencies of plasmid transfer were similar after 3- and 16-h mating, although the TFs to P. resinovorans were lower at 16 h than at 3 h. Higher growth rates of donor cells than of transconjugant cells during the mating procedure might be responsible for the observed lower TFs at 16 h than at 3 h. Therefore, we concluded that the longer mating time is not suitable for comparison of TFs. Since 1 h was too short a time to perform experiments in triplicate, we adopted 3 h as the optimal mating time for all mating experiments in this study. In addition, we also counted the cell number after the mating assays and confirmed that there was no effect of the viability or growth of each strain on the TF of plasmids during the 3-h mating assay procedure (see Table S1 in the supplemental material).

10.1128/mSphere.00490-18.2 FIG S1 Transfer frequency (TF) of each plasmid in 1:1 and 1:2 liquid mating assays. Plasmid-harboring (A) P. putida or (B) P. resinovorans was used as the donor, and P. putida or P. resinovorans (or both) was used as a recipient in 1:1 (or 1:2) mating assays. Mating assays were performed for 1, 3, and 16 h. Experiments were performed in triplicate for the 3- and 16-h matings, and bars show mean numbers of TFs (transconjugants/donor) calculated from triplicate spots of serially diluted mixtures. White diamonds show the frequencies calculated from each spot. For 1-h mating, experiments were performed at a single line three times and bars show results representative of technical triplicate experiments. White bars show the TFs of the plasmid to P. putida, and black bars show those to P. resinovorans. Download FIG S1, TIF file, 0.7 MB. Copyright © 2018 Sakuda et al. 2018 Sakuda et al. This content is distributed under the terms of the Creative Commons Attribution 4.0 International license.

10.1128/mSphere.00490-18.4 TABLE S1 Cell numbers of donors, recipients, and transconjugants (CFU per milliliter). Download Table S1, DOCX file, 0.03 MB. Copyright © 2018 Sakuda et al. 2018 Sakuda et al. This content is distributed under the terms of the Creative Commons Attribution 4.0 International license.

**FIG 1:**
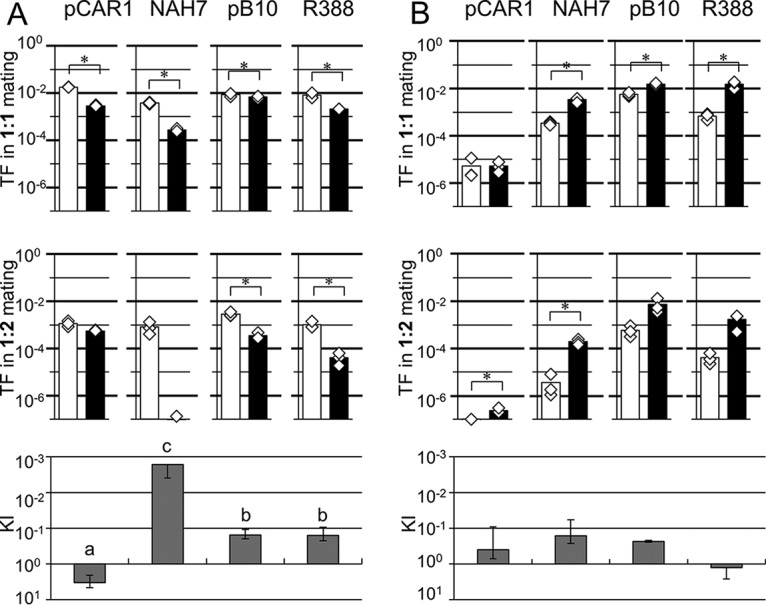
Transfer frequency (TF) of each plasmid in the 1:1 mating (upper panels) and 1:2 mating (lower panels) assays of liquid mating. Plasmid-harboring strains of Pseudomonas putida (A) or P. resinovorans (B) were used as donors. As the recipient strain(s), P. putida (or P. resinovorans) and both strains were used in 1:1 and 1:2 mating assays, respectively. Cell mixtures were incubated in microtubes containing LB for 3 h at 30°C to allow mating. Bars show the mean TFs (transconjugants/donor) calculated from triplicate assays (shown by white diamonds). White bars show TFs of plasmids to P. putida, and black bars show TFs of plasmids to P. resinovorans. All experiments were performed twice, and their reproducibility was confirmed. Asterisks indicate significant differences between two conditions as assessed by Student's *t* test (*P < *0.05) (*n* = 3). Kin indices (KIs) were calculated according to the equation [ratio in 1:2 mating (*r*_1:2_)]/[ratio in 1:1 mating (*r*_1:1_)], where *r*_1:2_ and *r*_1:1_ represent the ratios of TFs to different species/ratios of TFs to the same species during 1:2 mating and the ratios of TFs to different species/ratios of TFs to the same species during 1:1 mating, respectively. Note that the *y* axis data are inverted. Different letters above the bars indicate significant differences (*P < *0.05 [Kruskal-Wallis test], *P < *0.05 [Conover-Iman test], *n* = 3).

The TFs of the plasmids during liquid mating using P. putida as the donor are shown in Fig. 1A. The TFs of plasmids for P. putida were higher than those for P. resinovorans during 1:1 mating. In 1:2 mating, although the TFs detected were generally lower than those detected in 1:1 mating, higher TFs were detected in P. putida as recipient than in P. resinovorans as recipient. In particular, transfer of NAH7 from P. putida to P. resinovorans was markedly reduced during 1:2 mating (Fig. 1A). To evaluate the recipient preference in 1:2 mating quantitatively, we defined the kin index (KI), which shows the effect of candidate recipients from the same species on TFs compared to the effect of candidate recipients from different species during 1:2 mating (Fig. 1 [see also Table S2]; for the statistical analyses, see Materials and Methods and Table S3). The KI was calculated according to the following equation, where *r*_1:2_ represents the ratios of TFs to different species/the ratios of TFs to the same species during 1:2 mating and *r*_1:1_ represents the ratios of TFs to different species/the ratios of TFs to the same species during 1:1 mating:

$$\text{KI}=r_{1:2}/r_{1:1}$$

10.1128/mSphere.00490-18.5 TABLE S2 Ratios of TFs and kin indices (KIs) of respective plasmids. Asterisks (*) indicate ratios of TFs to different species/ratios of TFs to the same species during 1:1 mating. Daggers (†) indicate ratios of TFs to different species/ratios of TFs to the same species during 1:2 mating. Double daggers (‡) indicate KIs, which were calculated by the following equation: KI = *r*_1:2_/*r*_1:1_. Different superscript letters (^a^, ^b^, ^c^) indicate significant differences between plasmids in statistical analysis (*P < *0.05 [Kruskal-Wallis test], *P < *0.05 [Conover-Iman test], *n *=* *3). Download Table S2, DOCX file, 0.02 MB. Copyright © 2018 Sakuda et al. 2018 Sakuda et al. This content is distributed under the terms of the Creative Commons Attribution 4.0 International license.

10.1128/mSphere.00490-18.6 TABLE S3 *P* values of multiple comparisons by Conover-Iman test for data in Table S2. Statistically significantly different values are shaded and marked with asterisks as follows: *, *P < *0.05; **, *P < *0.01. Download Table S3, DOCX file, 0.02 MB. Copyright © 2018 Sakuda et al. 2018 Sakuda et al. This content is distributed under the terms of the Creative Commons Attribution 4.0 International license.

As shown in Fig. 1 and Table S2, the ratios of the TFs to different recipients/the TFs to the same recipient for pCAR1 were similar during 1:1 and 1:2 liquid mating experiments using the P. putida strain as the donor. On the basis of this result, it could be concluded that the coexistence of P. putida (same species) as a candidate recipient in the same location had no or negligible effect on the TF of pCAR1 to P. resinovorans (different species) during 1:2 mating. In contrast, there were larger differences between those ratios when the other plasmids were used, and the KIs were less than 1. The comparison of KIs among the four plasmids clearly showed that transfer of NAH7 to a different species recipient was reduced dramatically by the presence of same species recipient (KI = 1.7E−03 ± 2.3E−03) (Table S2).

Next, the TFs of the four plasmids using P. resinovorans as a donor during liquid mating were assessed (Fig. 1B). NAH7, pB10, and R388 were transferred more frequently to P. resinovorans than to P. putida during 1:1 mating. Similar tendencies were observed in 1:2 mating, although there were no statistically significant differences between the TFs of pB10 and R388 to P. putida and to P. resinovorans. The TFs of pCAR1 from donor P. resinovorans to each recipient were comparable during 1:1 mating, although the values were very low. In the case of 1:2 mating, the values were near the detection limit. As shown in Table S2, the KIs of pCAR1, NAH7, and pB10 were 4.0E−01 ± 3.1E−01, 1.6E−01 ± 1.0E−01, and 2.3E−01 ± 1.5E−02, respectively, suggesting that the TFs of these three plasmids from P. resinovorans to P. putida were slightly affected by coexisting P. resinovorans during 1:2 mating, although there were no statistically significant differences between the data from the four plasmids (Fig. 1; see also Table S2 and Table S3).

### Filter mating experiments.

**FIG 2:**
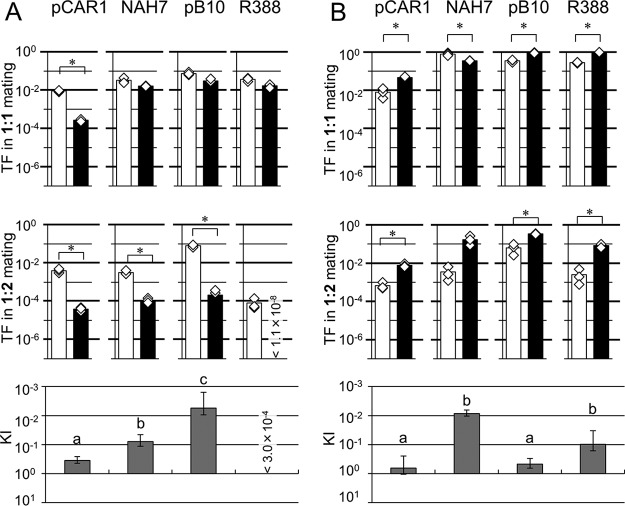
Transfer frequency (TF) of each plasmid in 1:1 mating (upper panels) and 1:2 mating (lower panels) assays of filter mating. Plasmid-harboring strains of Pseudomonas putida (A) or P. resinovorans (B) were used as donors. As recipient strain(s), P. putida (or P. resinovorans) and both strains were used in 1:1 and 1:2 mating assays, respectively. Cell mixtures were incubated for mating on a solid-agar LB plate surface for 3 h at 30°C. Bars show mean TFs (transconjugants/donor) calculated from triplicate assays (shown by open diamonds). White bars show the TF of the plasmid to P. putida, and black bars show the TF of the plasmid to P. resinovorans. All experiments were performed twice, and their reproducibility was confirmed. Asterisks indicate significant differences between two conditions as assessed by Student's *t* test (*P < *0.05) (*n* = 3). Kin indices (KIs) were calculated according to the equation [ratio in 1:2 mating (*r*_1:2_)]/ratio in 1:1 mating (*r*_1:1_)], where *r*_1:2_ and *r*_1:1_ represent the ratios of TFs to different species/ratios of TFs to the same species during 1:2 mating and the ratios of TFs to different species/ratios of TFs to the same species during 1:1 mating, respectively. Note that the *y* axis data are inverted. Different letters above the bars indicate significant differences (*P < *0.05 [Kruskal-Wallis test], *P < *0.05 [Conover-Iman test], *n* = 3).

Plasmid behavior can change between liquid and filter mating (30). It is likely that cell motility on a solid surface is restricted compared with that in liquid. Therefore, we assessed the effects of a coexisting candidate recipient in the 1:2 mating assay on a solid surface using the same combinations of donor/recipient strains and plasmids. It is known that plasmids which make short rigid pili are transferred with higher frequency in filter mating than in liquid (36). Consistent with these findings, the TFs of NAH7, pB10, and R388, which make short rigid pili, on solid surfaces were higher than those seen in the liquid mating experiments (Fig. 2). The results obtained using P. putida as a donor of the four plasmids are shown in Fig. 2A. The TFs of NAH7, pB10, and R388 to P. resinovorans were slightly lower during 1:1 mating than the corresponding TFs to P. putida. However, the TFs of these three plasmids to P. resinovorans were markedly lower than the TFs to P. putida during 1:2 mating and the KIs were <0.1, suggesting that transfer from P. putida to P. resinovorans was highly affected by the presence of coexisting P. putida cells (Fig. 2; see also Table S2). Notably, transfer of R388 to P. resinovorans was detected at a frequency of 1.7 × 10^−2^ during 1:1 mating, but the TF of this plasmid to P. resinovorans was reduced to below the detection limit during 1:2 mating. On the other hand, pCAR1 was preferably transferred to P. putida in both the 1:1 and 1:2 mating experiments, and the KI was 3.5E−01 ± 9.3E−02. These findings suggested that the effect of the presence of coexisting P. putida on pCAR1 transfer was lower than that seen with the other three plasmids and that this tendency was similarly seen in the liquid mating experiments (Fig. 1A; see also Table S2).

Next, P. resinovorans was used as the donor, and the results are shown in Fig. 2B. The TFs from P. resinovorans seen under the filter mating conditions were generally higher than those obtained when P. putida was used as the donor (Fig. 2A). Although the TF of NAH7 to P. putida was slightly higher than that to P. resinovorans during 1:1 mating, the frequency of transfer of the plasmids to P. resinovorans was generally higher than that to P. putida in both the 1:1 and 1:2 mating combinations. The KIs of pCAR1 and pB10 were 6.5E−01 ± 4.0E−01 and 4.7E−01 ± 1.7E−01, respectively, which are statistically significantly higher values than those seen with NAH7 and R388 (Fig. 2; see also Table S2 and Table S3). These results suggested that the effects of the presence of coexisting P. resinovorans on the conjugal transfer of pCAR1 and pB10 from P. resinovorans to P. putida were lower than those seen with NAH7 and R388.

### Effect of cell aggregation.

**FIG 3:**
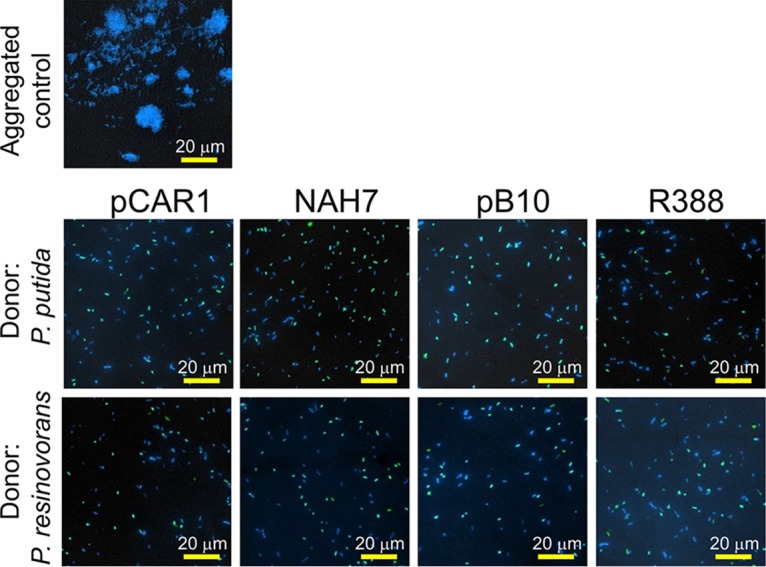
Microscopy of the donor and recipient strains in 1:2 liquid mating culture. Plasmid-harboring strain P. putida SM1443 or strain P. resinovorans CA10L was used as the donor, while P. putida KT2440RGdr and P. resinovorans CA10dm4RGgfp were used as recipients. P. putida KT2440RGdr was used as a positive control for aggregation, since that strain has been shown empirically to aggregate in medium containing succinate as the sole carbon source. Cells were stained with 50 μg/ml 4’,6’-diamidino-2-phenylindole (DAPI; blue), where P. resinovorans CA10dm4RGgfp cells exhibited green fluorescence. An aliquot (2 µl) of each mating culture was observed with fluorescence microscopy (BX53; Olympus). The resulting images were analyzed using DP2-BSW software (Olympus). Scale bar, 20 µm.

Because cell aggregation may enhance plasmid transfer (37), we assessed whether aggregation of cells of the same species donor and recipient strains occurred. Cells were subjected to 1:2 liquid mating conditions and were observed by fluorescence microscopy. As shown in Fig. 3, when P. putida was used as the donor strain, the cells of two recipient candidates, P. putida (blue) and P. resinovorans (green), showed a free-living state, and no large aggregation of cells was detected in the mating mixtures for each plasmid. Similar results were observed when P. resinovorans was used as the donor. These results were markedly different from those seen with the aggregated control sample. Although we cannot rule out the possible existence of small aggregations of 2 to 3 cells in the mating mixture, these observations suggested that formation of large cell aggregates was not the cause of the effect of the presence of coexisting recipients on plasmid transfer.

### Effect of culture supernatant.

**FIG 4:**
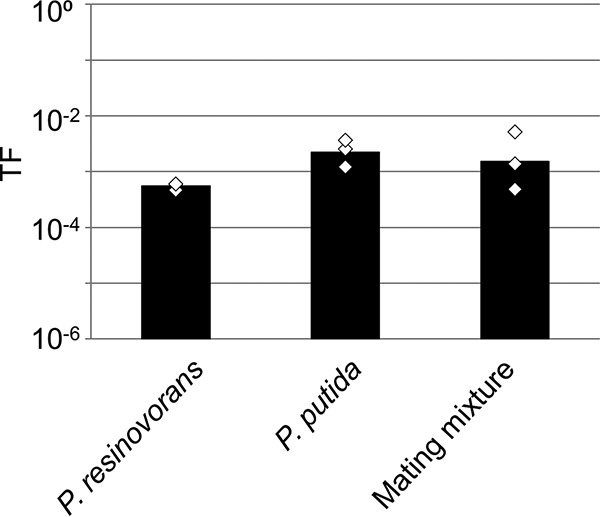
Transfer frequency (TF) of NAH7 during liquid mating with culture supernatant. Pseudomonas putida was used as the donor, and P. resinovorans was used as the recipient. An aliquot (400 µl) of the filtered culture supernatant of P. resinovorans or of P. putida or of a 3-h mating culture of NAH7-harboring P. putida and P. putida was added to the mixture of collected donor and recipient cells. Bars show mean TFs (transconjugants/donor) calculated from triplicate assays (shown by open diamonds). All experiments were performed twice to confirm reproducibility.

We evaluated whether the candidate recipient strain(s) secreted a substance(s) into the culture supernatant that affected plasmid transfer. In this study, we added the supernatant of a P. putida culture or of the mating mixture of NAH7-harboring P. putida and P. putida to the cell mixture of NAH7-harboring P. putida and P. resinovorans (as a 1:1 mating experiment). If the compound(s) secreted into the culture supernatant affected NAH7 conjugation to P. resinovorans, the TF of NAH7 to P. resinovorans would be decreased by adding the supernatants. However, the TF of NAH7 was not affected, as shown in Fig. 4. This result clearly showed that no compound affecting the conjugation of NAH7 to P. resinovorans was secreted into the P. putida culture supernatant or into that of the mating mixture of NAH7-harboring P. putida and P. putida.

## DISCUSSION

Our results suggest that plasmids were transferred generally more frequently to the same species recipient than to different species in 1:1 combinations under the liquid and filter mating conditions. The genome modification system of the host, such as methylation, a restriction enzyme system, and a CRISPR-Cas system, might affect plasmid behavior under 1:1 mating condition. The presence of the same species recipient candidate affected plasmid transfer to different species during 1:2 mating, and the ratio of TF to different species were lower in most combinations of donor/recipient strains and plasmids. This tendency was clearly shown by the fact that the KIs detected in almost all experiments were <1 (Fig. 1 and 2; see also Table S2 in the supplemental material), suggesting common mechanisms underlying the plasmid behavior in multiple recipients. Notably, we used only two *Pseudomonas* strains as hosts in this study. Therefore, it will be necessary to confirm this phenomenon in other host strains and with other host/plasmid combinations.

It is noteworthy that the KIs of NAH7 transfer from P. putida during liquid mating (Fig. 1A) and of transfer of R388 from P. putida during filter mating (Fig. 2A) were much lower than were seen with the other strains, suggesting that the presence of the same species strain severely interfered with conjugation to a different species strain in these combinations. The data may also suggest that there are unknown factors that enhance the effect of the presence of the same species strain for specific plasmids under specific conditions. In contrast, the KIs of pCAR1 were higher than those of the other plasmids for most combinations of donor/recipient strains (Fig. 1 and 2; see also Table S2), although there were no statistically significant differences between KIs for each plasmid using P. resinovorans as the donor in liquid mating. In particular, the KI of pCAR1 conjugation from P. putida under liquid mating conditions was >1 (Fig. 1; see also Table S2), suggesting that the transfer machinery encoded on pCAR1 or the stability of donor-recipient mating pair formation (MPF) can reduce the effect of the presence of other coexisting strains. Whether or not other IncP-7 plasmids would also have similar characteristics in 1:2 mating experiments should be confirmed.

We explored the relaxase encoded on the plasmids used in this study (mobility [MOB] classification [38]), where NAH7 and R388 were classified as MOB_F_, pB10 was classified as MOB_P_, and pCAR1 was classified as MOB_H_. Both MOB_F_ and MOB_P_ have the “3H” motif, and their structures are similar, but MOB_H_ has the “HD hydrolase” motif (38). These differences might affect plasmid behavior, including the smaller effect of coexisting strains on the pCAR1 transfer (Fig. 1 and 2; see also Table S2). Furthermore, the pilus types of plasmids have been classified on the basis of the proteins used to form mating pairs; NAH7, pB10, and R388 encode MPF_T_, which forms short rigid pili as described above, whereas pCAR1 encodes MPF_F_, which forms long flexible pili (39). Because pili could initiate contact with the recipient cell during plasmid transfer, these differences in pili might also be the reason for the distinct forms of plasmid behavior in multiple recipients. To study the effects of MOB/MPF-type relaxase, we plan to use other plasmids belonging to the same or other MOB/MPF types to attempt to construct chimera plasmids for further analysis.

We have succeeded in revealing that there is a novel but unknown mechanism that determines the fate of conjugation by selecting recipient cells among the bacterial consortia. The phenomena observed in this study cannot be explained by factors reported in previous studies, which were found in a mating assay performed only with one strain as a donor and another strain as a recipient. We have not succeeded in understanding the molecular mechanisms or clarified whether the factor(s) involved in these phenomena is encoded on the plasmid or on the host or on both. However, further forthcoming analyses might close the gap between laboratory conditions and actual environmental conditions in the study of plasmid behavior.

## MATERIALS AND METHODS

### Bacterial strains, media, and culture conditions.

The bacterial strains and plasmids used in this study are listed in Table 1. E. coli and Pseudomonas strains were grown overnight in lysogeny broth (LB) (40) containing the appropriate antibiotics for selection at 37°C and 30°C, respectively. Antibiotics were used at final concentrations of 50 μg ml^−1^ for kanamycin (Km), 30 μg ml^−1^ for gentamicin (Gm), 25 μg ml^−1^ for rifampin (Rif), 100 μg ml^−1^ for ampicillin (Ap), and 12.5 μg ml^−1^ for tetracycline (Tc). Solid media were prepared by adding 1.6% (wt/vol) agar to liquid LB medium. Derivative plasmids of pCAR1, NAH7, pB10, and R388 (pCAR1::*rfp* [31], NAH7K2 [41], pB10::*rfp* [42], and R388::*rfp* [30], respectively) were used in this study to select transconjugants with respect to Km resistance. P. resinovorans strains harboring NAH7K2, pB10::*rfp*, or R388::*rfp* were constructed using a method similar to a previously described method (32). Each plasmid was transferred from P. putida SM1443 into P. resinovorans CA10L, with Km resistance and Tc resistance as the selection markers. P. putida KT2440RGdr and P. resinovorans CA10dm4RGgfp were constructed according to the following method. The P_A1/04/03_-*DsRed* gene cassette was inserted into the chromosome of the P. putida KT2440 spontaneous Rif^r^ strain by filter mating with E. coli K-12 [miniTn*7*(Gm)P_A1/04/03_*DeRedExpress*-a] (43), E. coli SM10/λ*pir*(pUX-BF13) (43), and E. coli DH5α(pRK2013) (44). Similarly, a P_A1/04/03_-*gfp* cassette was inserted into the chromosome of the P. resinovorans CA10dm4 spontaneous Rif^r^ strain by filter mating with E. coli K-12 [miniTn*7*(Gm)P_A1/04/03_*gfp*-a] (43), E. coli SM10/λ*pir*(pUX-BF13) (43), and E. coli DH5α(pRK2013) (44). Each cassette was transposed into the chromosomal *att*Tn*7* site located downstream of the *glmS* gene.

**TABLE 1:** Bacterial strains and plasmids used in this study

Bacterial strain or plasmid	Relevant characteristic(s)	Source or reference
Bacterial strains		
Escherichia coli DH5α	*F*^-^ f80d *lac*ZDM15 Δ(*lac*ZYA-*argF*)*U169 endA1 recA1 hsdR17*(r_K_^−^ m_K_^+^) *deoR thi-1* *supE44* λ *gyrA96 relA1*	Toyobo
Escherichia coli SM10/*λpir*	*thi-1 thr-1 leu-6 tonA21 lacY1 supE44 recA* chromosomal RP4-2 [Tc^r^::Mu Km^r^::Tn*7*] λ*pir*	45
Pseudomonas putida KT2440RGdr	Derivative strain of KT2440, spontaneous Rif^r^ with introduced Gm^r^ and *DsRed*	This study
Pseudomonas putida SM1443	Derivative strain of KT2440 with introduced *lacI*^q^ cassette inserted into the chromosome	46
Pseudomonas putida SM1443(pB10::*rfp*)	SM1443 carrying pB10::*rfp*, Km^r^	42
Pseudomonas putida SM1443(pCAR1::*rfp*)	SM1443 carrying pCAR1::*rfp*, Km^r^	31
Pseudomonas putida SM1443(NAH7K2)	SM1443 carrying NAH7K2, Km^r^	30
Pseudomonas putida SM1443(R388::*rfp*)	SM1443 carrying R388::*rfp*, Km^r^	30
Pseudomonas resinovorans CA10dm4RGgfp	Derivative strain of CA10dm4, spontaneous Rif^r^, with introduced Gm^r^ gene and *gfp*	This study
Pseudomonas resinovorans CA10L	Derivative strain of CA10dm4 with introduced *lac*I^q^ cassette and Tc^r^ gene inserted into the chromosome	32
Pseudomonas resinovorans CA10L(pB10::*rfp*)	CA10dm4L carrying pB10::*rfp*, Km^r^	This study
Pseudomonas resinovorans CA10L(pCAR1::*rfp*)	CA10dm4L carrying pCAR1::*rfp*, Km^r^	32
Pseudomonas resinovorans CA10L(NAH7K2)	CA10dm4L carrying NAH7K2, Km^r^	This study
Pseudomonas resinovorans CA10L(R388::*rfp*)	CA10dm4L carrying R388::*rfp*, Km^r^	This study

Plasmids		
pB10::*rfp*	Antibiotic resistance plasmid, IncP-1 group, with Km^r^ gene and *rfp* cassette	42
pCAR1::*rfp*	Carbazole-degradative plasmid, IncP-7, with Km^r^ gene and *rfp* cassette	31
NAH7K2	Naphthalene-degradative plasmid, IncP-9 group, with Km^r^ gene cassette	41
R388::*rfp*	Antibiotic resistance plasmid, IncW group, with Km^r^ gene and *rfp* cassette	30
pRK2013	Helper plasmid for mobilization of non-self-transmissible plasmid, ColE1 replicon, Km^r^	44
MiniTn7(Gm)P_A1/04/03_*DeRedExpress*-a	pMB9 replicon, mini-Tn*7* vector carrying in its NotI site P_A1/04/03_*DeRedExpress*, Gm^r^	43
MiniTn7(Gm)P_A1/04/03_*gfp*-a	pMB9 replicon, mini-Tn*7* vector carrying in its NotI site P_A1/04/03_*gfp*, Gm^r^	43
pUX-BF13	Helper plasmid containing Tn*7* transposition functions, R6K replicon, Ap^r^	47

### Mating assay.

For mating assays using P. putida strains as donors, P. putida SM1443(pCAR1::*rfp*) (31), P. putida SM1443(NAH7K2) (30), P. putida SM1443(pB10::*rfp*) (38), and P. putida SM1443(R388::*rfp*) (30) were used as donors of pCAR1::*rfp*, NAH7K2, pB10::*rfp*, and R388::*rfp*, respectively. For mating assays using P. resinovorans strains as donors, P. resinovorans CA10L(pCAR1::*rfp*), P. resinovorans CA10L(NAH7K2), P. resinovorans CA10L(pB10::*rfp*), and P. resinovorans CA10L(R388::*rfp*) were used as donors of pCAR1::*rfp*, NAH7K2, pB10::*rfp*, and R388::*rfp*, respectively. Strains P. putida KT2440RGdr and P. resinovorans CA10dm4RGgfp were used as recipients. Overnight cultures of donor and recipient cells were harvested and washed with fresh LB. The resulting cells were suspended in fresh LB to an optical density at 600 nm (OD_600_) of 2 × 10^−1^ for the donor and 2 × 10^0^ for the recipient. Equal volumes (200 μl) of donor and recipient cell suspensions were mixed for 1:1 mating. A 200-μl aliquot of the donor cell suspension and 100 µl of each recipient cell suspension were mixed for 1:2 mating. Donor and recipient cells were mixed in 2-ml microtubes sealed with a gas-permeable adhesive seal (Thermo Fisher Scientific, Waltham, MA, USA) and incubated for 1, 3, or 16 h at 30°C for liquid mating. A mixture of donor and recipient cells was transferred onto a 0.22-μm-pore-size membrane filter (Millipore, Billerica, MA, USA) using glass microanalysis filter holders and filtering flasks (Millipore) for filter mating. Each filter was placed on an LB agar plate and incubated at 30°C for 1, 3, or 16 h. After incubation, 10 or 100 μl of a diluted mixture from each tube was spotted or spread on selected agar plates. The number of donor cells was calculated by determination of the number of CFUs grown on Km-added agar plates. While the transconjugants could also be grown on the Km-added agar plates, the numbers of transconjugants were completely different from the number of donors (usually more than 10 times lower than the number of donors), so we ignored them, in accordance with a previous study (30). The number of recipient cells was similarly calculated by determination of the number of CFUs grown on Rif- and Gm-added agar plates, while the transconjugants could also be grown on this plate, and we ignored them also. The number of transconjugant cells was calculated by determination of the number of CFUs grown on Km-, Rif-, and Gm-added agar plate. The green fluorescent protein (GFP) was used in the 1:2 mating assay to distinguish the two recipient strains. The GFP fluorescence of colonies was detected by the use of a Dark Reader DR46B Transilluminator (Clare Chemical Research, Dolores, CO, USA). The *DsRed* gene was inserted together with the Gm resistance gene, and we used Gm resistance only as a marker of recipients in this study. We did not use the DsRed florescence as a marker because of the slow expression and weak fluorescence of DsRed. We verified that the method used to distinguish the two strains using GFP was correct by colony hybridization (see Text S1 and Fig. S2 in the supplemental material). The levels of donor, recipient, and transconjugant cells seen after the mating assays are shown in Table S1 in the supplemental material. TFs were calculated by dividing the CFU per milliliter of the transconjugant cells by the CFU per milliliter of the donor cells. All experiments were performed at least twice.

10.1128/mSphere.00490-18.1 TEXT S1 Confirmation of the GFP correlation with the two strains. Download Text S1, DOCX file, 0.04 MB. Copyright © 2018 Sakuda et al. 2018 Sakuda et al. This content is distributed under the terms of the Creative Commons Attribution 4.0 International license.

10.1128/mSphere.00490-18.3 FIG S2 Fluorescence imaging and colony hybridization for colonies on selected agar plates of transconjugants. P. putida was used as the donor and P. putida and P. resinovorans as the recipients in the 1:2 mating assay. (A to D) Green fluorescent protein (GFP) fluorescence of colonies on a selected agar plate of transconjugants (A) and membranes of colony hybridization (B to D). The GFP fluorescence of colonies was detected with a Dark Reader DR46B Transilluminator (Clare Chemical Research). The membranes were hybridized using different oligonucleotide probes. The probes were prepared from the 0.8-kb fragment of the *repA* gene on pCAR1 (B), the 1.0-kb fragment of the *parI* gene on the P. putida KT2440 chromosome (C), and the 0.5-kb fragment of the PCA10_13490 gene on the P. resinovorans CA10dm4 chromosome (D), as specific probes for the pCAR1 and recipient strains. The inset shows a magnified image of the square area. The colonies with GFP fluorescence (arrows) were colonies of P. resinovorans harboring the plasmids, and the colonies without GFP fluorescence were colonies of P. putida harboring the plasmids. The experiments were performed twice. Download FIG S2, TIF file, 1.2 MB. Copyright © 2018 Sakuda et al. 2018 Sakuda et al. This content is distributed under the terms of the Creative Commons Attribution 4.0 International license.

### Statistical analyses.

The data used to determine the effect of the different recipients on the conjugation frequency of the different plasmids were assessed using Student’s *t* tests (*P < *0.05). Differences in the KIs of each plasmid in liquid or filter mating assay were analyzed using the Kruskal-Wallis test (*P < *0.05). As a result, the *P* values corresponding to the results of comparisons of liquid mating using P. putida as the donor, liquid mating using P. resinovorans as the donor, filter mating using P. putida as the donor, and filter mating using P. resinovorans as the donor were calculated as 0.02451, 0.516, 0.01723, and 0.02488 from Table S2, respectively. Among these, multiple comparisons were performed for the data set with *P* values of *<*0.05 by the Conover-Iman test (i.e., liquid mating using P. putida as the donor, filter mating using P. putida as the donor, and filter mating using P. resinovorans as the donor). The results are shown in Table S3.

### Cell aggregation.

Plasmid-harboring strains of P. putida SM1443 or P. resinovorans CA10L were used as donors, while P. putida KT2440RGdr and P. resinovorans CA10dm4RGgfp were used as the two recipient strains. The 1:2 mating mixtures were prepared as described under “Mating assay” above and were incubated in 2-ml microcentrifuge tubes at 30°C for 3 h. After the incubation, each mating culture was stained with 50 μg ml^−1^ DAPI (4’,6’-diamidino-2-phenylindole) for 15 min at 25°C and 2 μl of each sample was observed with fluorescence microscopy (BX53; Olympus, Tokyo, Japan). The resulting images were analyzed using DP2-BSW software (Olympus). The recipient (P. putida KT2440RGdr) was grown in the medium with succinate as the sole carbon source at 30°C for 4 h to act as a positive control for aggregation.

### Mating assay with culture supernatant.

Culture supernatants were prepared by centrifuging the cell cultures (15,000 rpm, 2 min, 25°C) and filtering the supernatants with 0.22-μm-pore-size filters (Millipore). A 400-μl aliquot of the filtered supernatants of P. resinovorans CA10dm4RGgfp or P. putida KT2440RGdr or of 3-h mating cultures of P. putida SM1443(NAH7K2) and P. putida KT2440RGdr was prepared. The mating mixtures of P. putida SM1443(NAH7K2) and P. resinovorans CA10dm4RGgfp were prepared as described under “Mating assay” above, and the cells were collected from the mating mixtures by centrifugation (15,000 rpm, 2 min, 25°C). After suspension of the cells in the culture supernatants, the mating mixtures were sealed in 2-ml microtubes with a gas-permeable adhesive seal (Thermo Fisher Scientific) and were incubated at 30°C for 3 h. TFs were calculated as described under “Mating assay” above.
