# Supplementary figures and images for: Conjugative Selectivity of Plasmids Is Affected by Coexisting Recipient Candidates
Source: mSphere. 2018 Dec 19;3(6):e00490-18. doi: 10.1128/mSphere.00490-18 (PMC6300686; doi:10.1128/mSphere.00490-18)

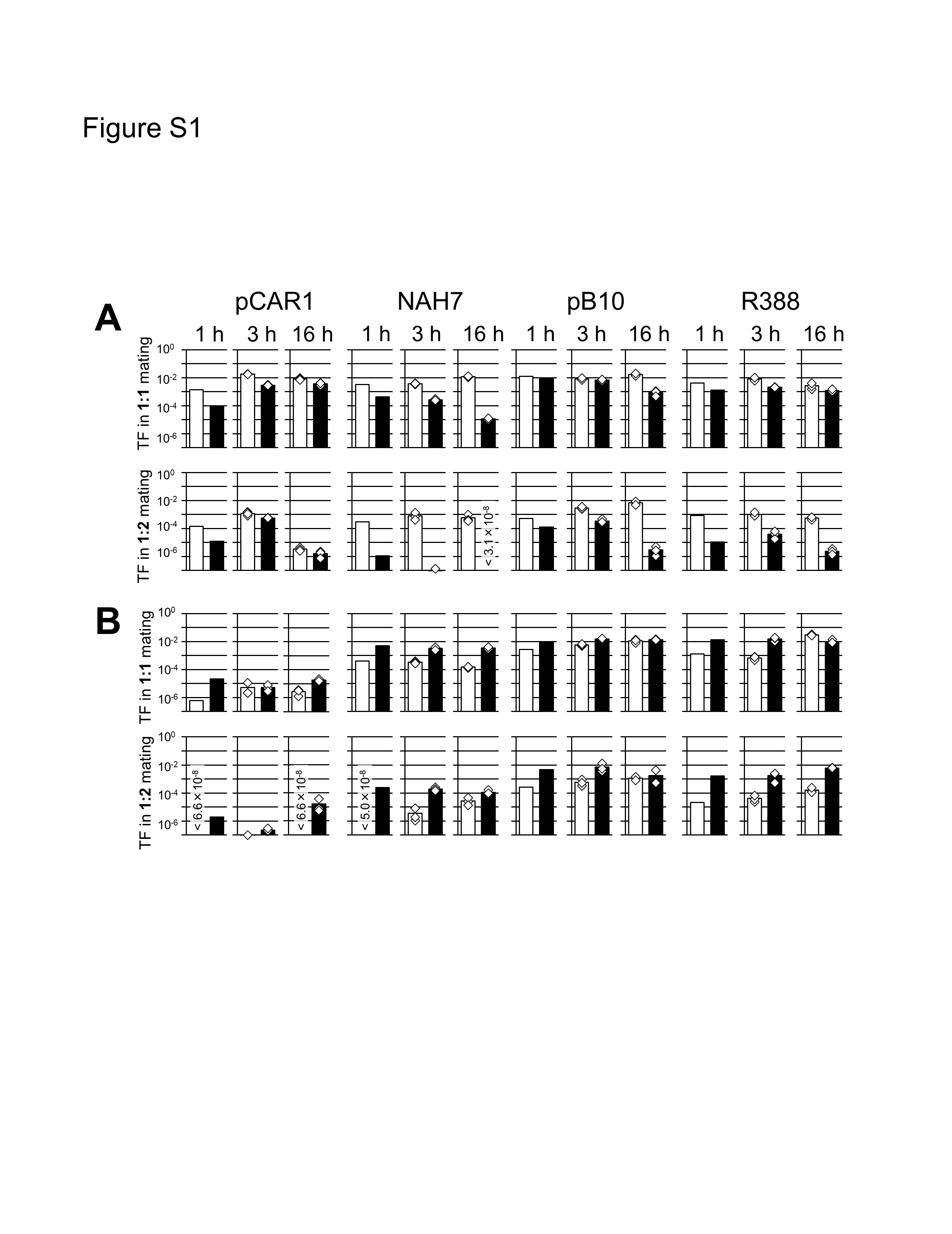

Supplement: FIG S1 [file sph006182730sf1.tif]

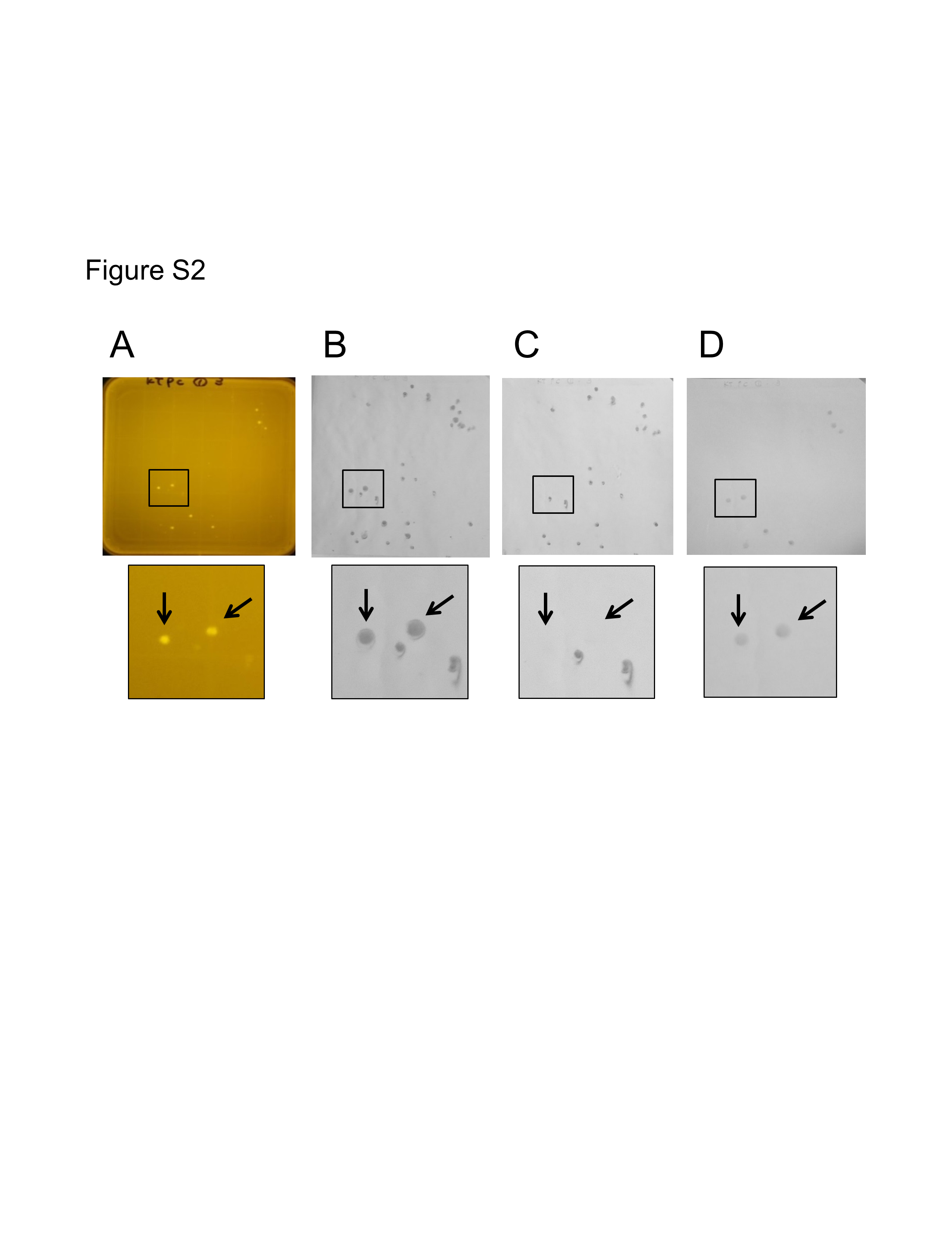

Supplement: FIG S2 [file sph006182730sf2.tif]
