# Supplementary material for: Conjugative Selectivity of Plasmids Is Affected by Coexisting Recipient Candidates
Source: mSphere. 2018 Dec 19;3(6):e00490-18. doi: 10.1128/mSphere.00490-18 (PMC6300686; doi:10.1128/mSphere.00490-18)
Supplement: TABLE S1 [file sph006182730st1.docx]

Table S1.

| **Liquid mating using *P. putida* as the donor** | | | | | | | |
| --- | --- | --- | --- | --- | --- | --- | --- |
|  | | Donor | | Recipient | | Transconjugant | |
| pCAR1 | 1:1 mating | *P. putida* | 6.7E+07 ± 5.7E+06 | *P. putida* | 6.3E+08 ± 8.9E+07 | *P. putida* | 1.2E+06 ± 8.3E+04 |
|  |  | *P. putida* | 6.2E+07 ± 2.6E+06 | *P. resinovorans* | 2.3E+09 ± 2.0E+08 | *P. resinovorans* | 1.7E+05 ± 2.3E+04 |
|  | 1:2 mating | *P. putida* | 5.9E+07 ± 5.3E+06 | *P. putida* | 3.5E+08 ± 4.6E+07 | *P. putida* | 6.7E+04 ± 1.0E+04 |
|  |  |  |  | *P. resinovorans* | 1.0E+09 ± 1.0E+08 | *P. resinovorans* | 3.3E+04 ± 5.7E+03 |
| NAH7 | 1:1 mating | *P. putida* | 6.9E+07 ± 3.7E+06 | *P. putida* | 1.1E+09 ± 3.1E+07 | *P. putida* | 2.6E+05 ± 5.7E+03 |
|  |  | *P. putida* | 8.4E+07 ± 9.6E+06 | *P. resinovorans* | 2.6E+09 ± 3.3E+08 | *P. resinovorans* | 2.2E+04 ± 1.5E+03 |
|  | 1:2 mating | *P. putida* | 7.3E+07 ± 3.8E+06 | *P. putida* | 2.2E+08 ± 3.9E+07 | *P. putida* | 5.8E+04 ± 2.4E+04 |
|  |  |  |  | *P. resinovorans* | 7.6E+08 ± 2.1E+08 | *P. resinovorans* | 3.3E+00 ± 4.7E+00 |
| pB10 | 1:1 mating | *P. putida* | 7.8E+07 ± 6.2E+06 | *P. putida* | 6.8E+08 ± 2.0E+07 | *P. putida* | 6.6E+05 ± 9.6E+04 |
|  |  | *P. putida* | 8.0E+07 ± 3.0E+06 | *P. resinovorans* | 2.5E+09 ± 2.4E+08 | *P. resinovorans* | 5.4E+05 ± 6.2E+04 |
|  | 1:2 mating | *P. putida* | 8.6E+07 ± 2.8E+06 | *P. putida* | 2.6E+08 ± 9.8E+07 | *P. putida* | 2.5E+05 ± 3.9E+04 |
|  |  |  |  | *P. resinovorans* | 1.1E+09 ± 2.7E+08 | *P. resinovorans* | 3.0E+04 ± 8.5E+03 |
| R388 | 1:1 mating | *P. putida* | 5.4E+07 ± 4.9E+06 | *P. putida* | 5.5E+08 ± 5.7E+07 | *P. putida* | 4.3E+05 ± 5.1E+04 |
|  |  | *P. putida* | 4.3E+07 ± 2.7E+06 | *P. resinovorans* | 2.2E+09 ± 9.4E+07 | *P. resinovorans* | 9.1E+04 ± 4.3E+03 |
|  | 1:2 mating | *P. putida* | 5.0E+07 ± 2.0E+06 | *P. putida* | 3.6E+08 ± 6.1E+07 | *P. putida* | 5.2E+04 ± 1.6E+04 |
|  |  |  |  | *P. resinovorans* | 1.2E+09 ± 8.2E+07 | *P. resinovorans* | 2.1E+03 ± 9.8E+02 |

Table S1. cont.

| **Liquid mating using *P. resinovorans* as the donor** | | | | | | | |
| --- | --- | --- | --- | --- | --- | --- | --- |
|  |  | Donor | | Recipient | | Transconjugant | |
| pCAR1 | 1:1 mating | *P. resinovorans* | 1.3E+08 ± 4.7E+07 | *P. putida* | 6.0E+08 ± 1.4E+07 | *P. putida* | 4.4E+02 ± 1.6E+02 |
|  |  | *P. resinovorans* | 1.7E+08 ± 1.2E+07 | *P. resinovorans* | 2.2E+09 ± 2.4E+08 | *P. resinovorans* | 8.3E+02 ± 2.7E+02 |
|  | 1:2 mating | *P. resinovorans* | 1.7E+08 ± 6.3E+07 | *P. putida* | 3.7E+08 ± 5.4E+07 | *P. putida* | 6.7E+00 ± 4.7E+00 |
|  |  |  |  | *P. resinovorans* | 1.0E+09 ± 6.7E+07 | *P. resinovorans* | 3.7E+01 ± 9.4E+00 |
| NAH7 | 1:1 mating | *P. resinovorans* | 1.9E+08 ± 3.0E+07 | *P. putida* | 1.3E+09 ± 4.6E+08 | *P. putida* | 6.2E+04 ± 5.4E+03 |
|  |  | *P. resinovorans* | 2.0E+08 ± 3.0E+07 | *P. resinovorans* | 2.6E+09 ± 1.6E+08 | *P. resinovorans* | 6.4E+05 ± 3.1E+04 |
|  | 1:2 mating | *P. resinovorans* | 1.7E+08 ± 9.8E+06 | *P. putida* | 2.4E+08 ± 4.9E+07 | *P. putida* | 6.0E+02 ± 5.0E+02 |
|  |  |  |  | *P. resinovorans* | 1.3E+08 ± 5.2E+07 | *P. resinovorans* | 3.3E+04 ± 6.3E+03 |
| pB10 | 1:1 mating | *P. resinovorans* | 8.8E+07 ± 2.5E+06 | *P. putida* | 5.9E+08 ± 2.9E+07 | *P. putida* | 5.0E+05 ± 6.7E+04 |
|  |  | *P. resinovorans* | 8.5E+07 ± 3.3E+06 | *P. resinovorans* | 2.5E+09 ± 2.6E+08 | *P. resinovorans* | 1.3E+06 ± 1.5E+05 |
|  | 1:2 mating | *P. resinovorans* | 8.3E+07 ± 8.2E+06 | *P. putida* | 2.5E+08 ± 6.8E+07 | *P. putida* | 4.8E+04 ± 1.8E+04 |
|  |  |  |  | *P. resinovorans* | 1.2E+09 ± 9.7E+07 | *P. resinovorans* | 5.9E+05 ± 3.0E+05 |
| R388 | 1:1 mating | *P. resinovorans* | 8.1E+07 ± 4.2E+06 | *P. putida* | 5.1E+08 ± 2.5E+07 | *P. putida* | 5.6E+04 ± 1.2E+04 |
|  |  | *P. resinovorans* | 7.3E+07 ± 1.1E+07 | *P. resinovorans* | 2.5E+09 ± 1.2E+08 | *P. resinovorans* | 1.1E+06 ± 1.5E+05 |
|  | 1:2 mating | *P. resinovorans* | 8.4E+07 ± 3.9E+06 | *P. putida* | 2.8E+08 ± 6.1E+07 | *P. putida* | 3.3E+03 ± 1.3E+03 |
|  |  |  |  | *P. resinovorans* | 1.4E+09 ± 9.3E+07 | *P. resinovorans* | 1.5E+05 ± 7.5E+04 |

Table S1. cont.

| **Filter mating using *P. putida* as the donor** | | | | | | | |
| --- | --- | --- | --- | --- | --- | --- | --- |
|  |  | Donor | | Recipient | | Transconjugant | |
| pCAR1 | 1:1 mating | *P. putida* | 3.1E+08 ± 5.8E+07 | *P. putida* | 7.6E+08 ± 8.8E+07 | *P. putida* | 2.9E+06 ± 6.7E+05 |
|  |  | *P. putida* | 2.7E+08 ± 4.2E+07 | *P. resinovorans* | 6.2E+08 ± 3.0E+07 | *P. resinovorans* | 7.2E+04 ± 7.2E+03 |
|  | 1:2 mating | *P. putida* | 2.7E+08 ± 8.7E+07 | *P. putida* | 3.9E+08 ± 6.6E+07 | *P. putida* | 1.1E+06 ± 4.7E+05 |
|  |  |  |  | *P. resinovorans* | 2.8E+07 ± 3.1E+06 | *P. resinovorans* | 1.0E+04 ± 2.2E+03 |
| NAH7 | 1:1 mating | *P. putida* | 2.2E+08 ± 4.2E+07 | *P. putida* | 7.5E+08 ± 3.7E+07 | *P. putida* | 6.8E+06 ± 4.9E+05 |
|  |  | *P. putida* | 2.3E+08 ± 2.3E+07 | *P. resinovorans* | 3.8E+09 ± 8.3E+07 | *P. resinovorans* | 3.8E+06 ± 4.0E+05 |
|  | 1:2 mating | *P. putida* | 2.3E+08 ± 5.2E+07 | *P. putida* | 4.3E+08 ± 1.5E+08 | *P. putida* | 7.7E+05 ± 3.1E+05 |
|  |  |  |  | *P. resinovorans* | 8.1E+07 ± 3.2E+07 | *P. resinovorans* | 2.5E+04 ± 1.9E+03 |
| pB10 | 1:1 mating | *P. putida* | 3.9E+08 ± 2.7E+07 | *P. putida* | 5.9E+08 ± 5.2E+07 | *P. putida* | 2.8E+07 ± 2.0E+06 |
|  |  | *P. putida* | 2.3E+08 ± 2.2E+07 | *P. resinovorans* | 5.0E+08 ± 1.0E+08 | *P. resinovorans* | 6.9E+06 ± 7.3E+05 |
|  | 1:2 mating | *P. putida* | 4.2E+08 ± 1.1E+07 | *P. putida* | 3.0E+08 ± 3.1E+07 | *P. putida* | 3.3E+07 ± 4.3E+06 |
|  |  |  |  | *P. resinovorans* | 2.6E+07 ± 4.2E+06 | *P. resinovorans* | 9.0E+04 ± 6.7E+04 |
| R388 | 1:1 mating | *P. putida* | 2.5E+08 ± 1.7E+07 | *P. putida* | 6.6E+08 ± 6.5E+07 | *P. putida* | 9.3E+06 ± 9.8E+05 |
|  |  | *P. putida* | 3.5E+08 ± 1.9E+07 | *P. resinovorans* | 6.3E+08 ± 6.6E+07 | *P. resinovorans* | 5.9E+06 ± 7.2E+05 |
|  | 1:2 mating | *P. putida* | 2.9E+08 ± 2.7E+06 | *P. putida* | 4.1E+08 ± 6.0E+07 | *P. putida* | 2.3E+04 ± 1.3E+04 |
|  |  |  |  | *P. resinovorans* | 3.4E+07 ± 8.8E+06 | *P. resinovorans* | 0.0E+00 ± 0.0E+00 |

Table S1. cont.

| **Filter mating using *P. resinovorans* as the donor** | | | | | | | |
| --- | --- | --- | --- | --- | --- | --- | --- |
|  |  | Donor | | Recipient | | Transconjugant | |
| pCAR1 | 1:1 mating | *P. resinovorans* | 9.9E+05 ± 2.3E+05 | *P. putida* | 1.8E+09 ± 2.5E+08 | *P. putida* | 7.9E+03 ± 4.3E+03 |
|  |  | *P. resinovorans* | 4.7E+06 ± 4.4E+05 | *P. resinovorans* | 4.7E+09 ± 3.1E+08 | *P. resinovorans* | 2.1E+05 ± 7.9E+03 |
|  | 1:2 mating | *P. resinovorans* | 5.8E+06 ± 1.9E+05 | *P. putida* | 7.5E+08 ± 3.3E+08 | *P. putida* | 3.8E+03 ± 1.2E+03 |
|  |  |  |  | *P. resinovorans* | 6.6E+08 ± 2.8E+08 | *P. resinovorans* | 4.4E+04 ± 7.1E+03 |
| NAH7 | 1:1 mating | *P. resinovorans* | 5.9E+06 ± 5.3E+05 | *P. putida* | 1.9E+09 ± 2.7E+07 | *P. putida* | 4.5E+06 ± 4.6E+05 |
|  |  | *P. resinovorans* | 4.8E+08 ± 4.2E+07 | *P. resinovorans* | 2.8E+09 ± 9.6E+07 | *P. resinovorans* | 1.7E+08 ± 1.9E+07 |
|  | 1:2 mating | *P. resinovorans* | 2.0E+07 ± 4.4E+06 | *P. putida* | 6.0E+08 ± 6.7E+07 | *P. putida* | 6.7E+04 ± 3.7E+04 |
|  |  |  |  | *P. resinovorans* | 1.5E+08 ± 1.8E+07 | *P. resinovorans* | 3.4E+06 ± 1.4E+06 |
| pB10 | 1:1 mating | *P. resinovorans* | 1.6E+08 ± 5.7E+06 | *P. putida* | 1.9E+08 ± 2.2E+07 | *P. putida* | 5.6E+07 ± 5.6E+06 |
|  |  | *P. resinovorans* | 1.1E+09 ± 3.0E+07 | *P. resinovorans* | 4.1E+09 ± 1.7E+08 | *P. resinovorans* | 9.7E+08 ± 5.7E+07 |
|  | 1:2 mating | *P. resinovorans* | 6.3E+08 ± 6.5E+07 | *P. putida* | 2.0E+08 ± 1.4E+08 | *P. putida* | 3.7E+07 ± 1.5E+07 |
|  |  |  |  | *P. resinovorans* | 6.5E+08 ± 3.9E+08 | *P. resinovorans* | 2.1E+08 ± 2.9E+07 |
| R388 | 1:1 mating | *P. resinovorans* | 2.4E+07 ± 7.2E+06 | *P. putida* | 5.6E+08 ± 5.8E+07 | *P. putida* | 6.5E+06 ± 2.2E+06 |
|  |  | *P. resinovorans* | 8.1E+08 ± 1.3E+08 | *P. resinovorans* | 4.2E+09 ± 2.7E+07 | *P. resinovorans* | 7.6E+08 ± 1.3E+08 |
|  | 1:2 mating | *P. resinovorans* | 2.4E+08 ± 3.5E+07 | *P. putida* | 2.2E+08 ± 3.5E+07 | *P. putida* | 5.8E+05 ± 3.4E+05 |
|  |  |  |  | *P. resinovorans* | 1.3E+08 ± 2.8E+07 | *P. resinovorans* | 2.1E+07 ± 5.4E+06 |
