# Supplementary material for: Conjugative Selectivity of Plasmids Is Affected by Coexisting Recipient Candidates
Source: mSphere. 2018 Dec 19;3(6):e00490-18. doi: 10.1128/mSphere.00490-18 (PMC6300686; doi:10.1128/mSphere.00490-18)
Supplement: TABLE S2 [file sph006182730st2.docx]

Table S2.

|  |  |  | pCAR1 | NAH7 | pB10 | R388 |
| --- | --- | --- | --- | --- | --- | --- |
| Liquid mating | Donor:  *P. putida* | r_1:1_^*^ | 1.6E-01 ± 1.1E-02 | 7.0E-02 ± 4.7E-03 | 8.0E-01 ± 3.6E-02 | 2.7E-01 ± 5.1E-02 |
|  |  | r_1:2_^†^ | 5.2E-01 ± 1.7E-01 | 1.1E-04 ± 1.5E-04 | 1.3E-01 ± 4.3E-02 | 3.9E-02 ± 1.2E-02 |
|  |  | KI^‡^ | 3.4E+00 ± 1.3E+00^a^ | 1.7E-03 ± 2.3E-03^c^ | 1.6E-01 ± 4.6E-02^b^ | 1.6E-01 ± 6.4E-02^b^ |
|  | Donor:  *P. resinovorans* | r_1:1_^*^ | 8.8E-01 ± 4.3E-01 | 1.0E-01 ± 1.0E-02 | 3.7E-01 ± 2.9E-02 | 5.0E-02 ± 2.5E-02 |
|  |  | r_1:2_^†^ | 2.2E-01 ± 1.6E-01 | 1.7E-02 ± 1.1E-02 | 8.7E-02 ± 1.2E-02 | 5.0E-02 ± 5.3E-02 |
|  |  | KI^‡^ | 4.0E-01 ± 3.1E-01 | 1.6E-01 ± 1.0E-01 | 2.3E-01 ± 1.5E-02 | 1.3E+00 ± 1.4E+00 |
| Filter mating | Donor:  *P. putida* | r_1:1_^*^ | 3.0E-02 ± 4.1E-03 | 5.3E-01 ± 1.1E-01 | 4.3E-01 ± 1.2E-01 | 4.8E-01 ± 1.4E-01 |
|  |  | r_1:2_^†^ | 1.1E-02 ± 3.6E-03 | 3.8E-02 ± 1.4E-02 | 2.8E-03 ± 2.0E-03 | < 1.4E-04 |
|  |  | KI^‡^ | 3.5E-01 ± 9.3E-02^a^ | 7.9E-02 ± 3.3E-02^b^ | 5.5E-03 ± 4.0E-03^c^ | < 3.0E-04 |
|  | Donor:  *P. resinovorans* | r_1:1_^*^ | 1.7E-01 ± 6.5E-02 | 2.2E+00 ± 2.7E-01 | 3.9E-01 ± 5.9E-02 | 3.0E-01 ± 1.5E-02 |
|  |  | r_1:2_^†^ | 8.5E-02 ± 1.5E-02 | 1.8E-02 ± 4.4E-03 | 1.9E-01 ± 9.0E-02 | 2.8E-02 ± 1.7E-02 |
|  |  | KI^‡^ | 6.5E-01 ± 4.0E-01^a^ | 8.4E-03 ± 2.0E-03^b^ | 4.7E-01 ± 1.7E-01^a^ | 9.7E-02 ± 6.4E-02^b^ |
