# Supplementary material for: Conjugative Selectivity of Plasmids Is Affected by Coexisting Recipient Candidates
Source: mSphere. 2018 Dec 19;3(6):e00490-18. doi: 10.1128/mSphere.00490-18 (PMC6300686; doi:10.1128/mSphere.00490-18)
Supplement: TABLE S3 [file sph006182730st3.docx]

Table S3.

| Liquid mating using *P. putida* as the donor | | | |
| --- | --- | --- | --- |
|  | 1: pCAR1 | 2: NAH7 | 3: pB10 |
| 2: NAH7 | 0.0008^**^ | - | - |
| 3: pB10 | 0.0380^*^ | 0.0380^*^ | - |
| 4: R388 | 0.0380^*^ | 0.0380^*^ | 0.8067 |
| Filter mating using *P. putida* as the donor | | | |
|  | 1: pCAR1 | 2: NAH7 | 3: pB10 |
| 2: NAH7 | 0.02496^*^ | - | - |
| 3: pB10 | 0.00059^**^ | 0.01708^*^ | - |
| 4: R388 | 0.00010^**^ | 0.00148^**^ | 0.06497 |
| Filter mating using *P. resinovorans* as the donor | | | |
|  | 1: pCAR1 | 2: NAH7 | 3: pB10 |
| 2: NAH7 | 0.0026^**^ | - | - |
| 3: pB10 | 0.8089 | 0.0029^**^ | - |
| 4: R388 | 0.0323^*^ | 0.1091 | 0.0351^*^ |
