# Supplementary material for: Conjugative Selectivity of Plasmids Is Affected by Coexisting Recipient Candidates
Source: mSphere. 2018 Dec 19;3(6):e00490-18. doi: 10.1128/mSphere.00490-18 (PMC6300686; doi:10.1128/mSphere.00490-18)
Supplement: TEXT S1 [file sph006182730s1.docx]

**Supplemental Text S1**

**Supplemental methods**

**Confirmation of the GFP correlation with the two strains**

We checked whether the method employed to distinguish the strains with GFP fluorescens was correctly to be *P. resinovorans* strains by colony hybridization. The probes for colony hybridization were prepared from the 0.8-kb fragment of *repA* gene on pCAR1, the 1.0-kb fragment of *parI* gene on *P. putida* KT2440 chromosome and the 0.5-kb fragment of PCA10_13490 gene on *P. resinovorans* CA10dm4 chromosome, as specific probes for pCAR1 and host strains. It was detected that the cell which had GFP fluorescence was plasmid-harbouring *P. resinovorans* strain and the cell without GFP fluorescence was plasmid-harbouring *P. putida* strain (Fig. S2). The hybridization was performed with a DIG High Prime DNA Labelling and Detection Starter Kit I (Roche Applied Science) according to the respective manufacturers’ instructions.
